# A prospective study of health care resource utilisation and selected costs of schizophrenia in France

**DOI:** 10.1186/1472-6963-12-269

**Published:** 2012-08-21

**Authors:** Emmanuelle Sarlon, Dirk Heider, Aurélie Millier, Jean-Michel Azorin, Hans-Helmut König, Karina Hansen, Matthias C Angermeyer, Samuel Aballéa, Mondher Toumi

**Affiliations:** 1 National Institute of Health and Medical Research, INSERM, U669, Paris, France; 2 University of Paris-Sud and University of Paris Descartes, UMR-S0669, Paris, France; 3 Department of Public Health, Hospital Center, GHPSO, Creil/Senlis, Oise, France; 4 Department of Medical Sociology and Health Economics, University Medical Center Hamburg-Eppendorf, Hamburg, Germany; 5 Institute of Social Medicine, Occupational Health and Public Health (ISAP), University of Leipzig, Leipzig, Germany; 6 Creativ-Ceutical, Paris, France; 7 Service Universitaire de Psychiatrie, CHU Ste-Marguerite, Marseille, France; 8 Lundbeck, Paris, France; 9 Center for Public Mental Health, Gösing am Wagram, Austria; 10 UCBL 1 - Chair of Market Access University Claude Bernard Lyon I, Decision Sciences & Health Policy, Lyon, 69622, France

**Keywords:** Psychiatric epidemiology, Schizophrenia, Cost of illness

## Abstract

**Background:**

Schizophrenia is among the most burdensome and costly illnesses worldwide. To estimate the cost of schizophrenia in France, a longitudinal study was carried out between 1998 and 2002. The main objective of this study was to describe and update the cost of schizophrenia in a longitudinal, representative sample of French patients. The second objective was to identify cost drivers in the treatment of schizophrenia.

**Methods:**

Based on a cohort of 288 French schizophrenic patients during 2 years of prospective follow-up, this study collected clinical, patient reported outcomes, quality of life, functioning, patient management, care giver involvement and resource utilisation data every 6 months. For each service, information was collected on the type of service, the frequency of attendance and type of intervention provided to the patient. Unit costs were based on available French databases. Mean service use and costs over the five time points were estimated using between-effects regression models.

**Results:**

In the total sample of 288 patients aged 18-64 years, the mean total cost (€ 3 534) was mainly accounted for by the cost of inpatient treatment (€ 1 390) and day care (€ 1 331). The estimate of the annual cost for direct medical health care for all French schizophrenic patients was € 1 581 million, including € 621 million for inpatient treatment and € 595 million for day care (77%). The costs for medication accounted for 16.1% of total annual costs. The remaining costs (6.9%) included visits to psychiatrists, general practitioners, other physicians and psychologists. The direct resource allocation showed inpatient treatment as the main direct cost. Unemployment was identified as a major indirect cost of schizophrenia treatment. Positive and depressive schizophrenia symptoms at baseline and relapse occurrence during the follow-up period were associated with a higher cost of treatment. Health satisfaction or negative symptoms of schizophrenia at baseline were associated with lower costs.

**Conclusion:**

Several cost drivers were identified. Based on the results obtained in France, we suggest further analysis of mechanisms that influence the service-specific costs for schizophrenia in other areas of the world.

## Background

Schizophrenia, which is a psychiatric disease, is associated with significant and long-lasting health, social and financial burden, not only for patients but also for families, other caregivers and the wider society. Although the lifetime prevalence has not increased (estimated at 0.5% in a recent systematic review [1]), schizophrenia remains among the most burdensome and costly illnesses worldwide. Considerable progress has been made during the last 10 years in the treatment of schizophrenia. Patients with schizophrenia, given the severity of the disorder, usually require long-term health care. Though the economic costs vary from country to country, all the cost-of-illness estimates highlight the heavy societal burden of schizophrenia [2].

In a cross-sectional study, Rouillon et al. [3] estimated the direct and indirect annual (in 1992) cost of medical management of schizophrenia in France to be Fr 12.4 billion ($US 2.34 billion) and Fr 5.2 billion ($US 0.97 billion), respectively.

The main objective of this study was to describe and update the cost of schizophrenia using a longitudinal, representative sample of French schizophrenic patients. The second objective was to identify cost drivers in schizophrenia treatment.

## Methods

### Study design

The data are from the European Schizophrenia Cohort (EuroSC), conducted in the UK, France, and Germany. A detailed description of the European Schizophrenia Cohort has been published earlier [4]. It is a naturalistic, 2-year follow-up of a schizophrenic patient cohort conducted in Islington and Leicestershire (UK), in Lille, Lyon and Marseille (France), and in Hemer, Heilbronn, Altenburg and Leipzig (Germany). The main objective of this cohort was to identify and describe the types of treatment and methods of care for people with schizophrenia and to correlate these with clinical outcomes, states of health, and quality of life. This project was conducted in accordance with the Declaration of Helsinki and French Good Clinical Practices [5,6]. The study protocol was approved by the Institution Review Board or the Ethic Committee responsible for each participating hospital or institution (Camden & Islington Community Mental Health NHS Trust Ethics Committee and Leicester University Committee for Research Ethics for all UK sites, Ethics Committee of the University of Leipzig for Germany, and Ethics Committee of the University of Aix-Marseille 2 for France). Written informed consent was obtained from each participant after the study details had been fully explained.

### Subjects

In France, mental health care is driven by a law defining 800 catchment areas (sectors). Each sector has around 70 000 inhabitants. As resources are unequally allocated between sectors, the strategy was to integrate ten adjacent sectors into a single sampling area. Three such integrated areas were selected. They were located in northern France (Lille), central France (Lyon and Clermont-Ferrand) and southern France (Marseille and Toulon). Each of these areas covered an urban centre of approximately one million inhabitants living in a city or in medium-sized towns.

Sampling was achieved by establishing a list of all mental patients in the areas, using information from the mental health services, and then sampling at random from those patients that were identified. Participants were selected to provide a representative sample of the patients treated in secondary psychiatric services in each catchment area. Roofless mental patients were excluded from the study, as well as people who had been hospitalised for the previous 12 months or were planning to move out of the area. If the participant withdrew consent at any time or if the participant was lost to follow-up, data collected up to this point were used in the analysis.

### Assessments

At baseline, the *Structured Clinical Interview for DSM-IV (SCID)*[7] was used to establish the diagnosis. The SCID, administered by a clinician, is a record of the presence or absence of each of the symptoms and disorders being considered for the current episode (past month) and lifetime occurrence. Socio-demographic characteristics and the previous course of the illness were assessed with the *Past History and Sociodemographic Description Schedule (PHSD)*[8,9]. The PHSD was adapted for use in the study. The instrument was used in the WHO Collaborative Study on the Assessment and Reduction of Psychiatric Disability, and our adaptation was based on the third draft from 1977.

Clinical assessment of each patient was made by an external professional. Each assessor was trained for instruments used in the questionnaire. Schizophrenic symptomatology was assessed by means of the *Positive and Negative Syndrome Scale (PANSS)*[10,11]. Inter-rater reliability was reported as adequate or good [12]. Depression was measured using the *Calgary Depression Scale for Schizophrenia (CDSS)*[13]. The scale is composed of nine questions, rated on a four-point scale. A global score was obtained by adding the values for each item [14]. For the assessment of subjective quality of life, Lehman’s *Quality of Life Interview (QoLI)*[15] was used. This measure is comprised of eight domains: living situation (three items), daily activities (four items), family (two items), social relations (three items), finances (three items), legal and safety issues (three items), health (three items), and work (three items). The answers are recorded on a seven-point Likert scale anchored by 1 = ‘terrible’ and 7 = ‘delighted’. Item scores for each domain were combined into a single measure by calculating the mean item score.

Information about the use of services during the six-month period preceding each assessment was collected by means of the *Malin System*[16]. The system covered hospital-based services, day clinic activities, outpatient physician and psychological services, and medications used by the patient. For each service, information was collected on the type of service, the frequency of attendance and type of intervention provided to the patient.

### Unit cost estimates

#### Health care costs

Unit costs for outpatient visits were based on the Tarif Conventionnel, which is a national database for health information [17]. Unit costs for inpatient and day clinic treatment were based on a tariff per hospital day fixed by the regional health law and given by expert information from the Ministry of Health in France. In French psychiatric hospital financing, real costs, such as Diagnosis Related Group rates for a general hospital, are not available. Medication prices were taken from an internet source for reimbursable medication based on actual prices [18]. This database was accessed online in October 2007. All other cost estimates are based on data sources for the years 1999 and 2000.

#### Cost of lost productivity

A large part of the global economic impact of schizophrenia stems from the difficulties encountered by patients in finding and keeping paid employment. The most important feature of indirect costs is the loss of productivity because of patient morbidity and mortality (i.e., loss of ability to work) [2].

Here, we assumed that unemployment in patients of working age was the main indirect cost. The monetary value of lost productivity was estimated according to the human capital method [19]. The number of unemployed individuals was combined with an average gross annual pay of € 21 492 (for full-time employees during the tax year 2007 according to Institut National de la Statistique et des Etudes Economiques (INSEE) data) [20].

#### Statistical analysis

Mean service use and costs over the five time points were estimated using between-effects regression models for every type of resource use surveyed; the temporal order of the repetitions was not taken into account. The Weighted Least Squares approach allowed us to make sure that patients with more time points were weighted higher than those with fewer time points (considering their data are less informative/valid). This procedure took into account the fact that a greater number of completed follow-ups per respondent implied higher validity of the estimated mean costs. The model is derived from a random effects model:

(1) $$y_{\mathrm{it}}=\alpha +x_{\mathrm{it}}\beta +\nu_{i}+\varepsilon_{\mathrm{it}}$$

for *i = 1,…, n,* and for each *i, t = 1,…, Ti* of which Ti occasions are actually observed. It is defined as:

(2) $${\bar{y}}_{i}=\alpha +{\bar{x}}_{i}\beta +\nu_{i}+{\bar{\varepsilon }}_{i}$$

where ${\bar{y}}_{i}=\sum_{t=1}^{T_{i}}y_{\mathrm{it}}/T_{i}$ and ${\bar{x}}_{i}$ is defined similarly.

As the number of observations (Ti) is different for each patient, WLS (weighted least squares) were adopted for estimation, and *Ti* served as an analytic weight [21]. Since the data were highly right-skewed, non-parametric bootstrapping with 4 000 replications was applied. The analyses were carried out using STATA 10 [22].

## Results

Overall, 288 patients aged 18-64 years were recruited for the study. Sample attrition resulted in 223 participants taking part in the second interview, 196 in the third, 173 in the fourth, and 138 in the final interview.

### Socio-demographic and clinical characteristics

As shown in Table 1, the sample consisted of mostly single (72%), male patients (69.7%), with a mean age of 39.6 years. Their mean number of years of education was 10 years, and 88.5% were unemployed. On average, the patients had been hospitalised six times before inclusion in the study. Overall, 17.4% of patients relapsed during the two-year follow-up period.

**Table 1:** Baseline socio-demographic and clinical characteristics of the sample (N = 288)

**Variable**	**Result**
**Gender - N (%)**	
Male	200 (69.7%)
Female	87 (30.3%)
**Age - mean (SD)**	**39.6 (10.3)**
**Age categories - N (%)**	
18-30	62 (21.5%)
31-50	181 (62.9%)
51-65	45 (15.6%)
**Years of education - mean (SD)**	10.1 (3.0)
**Education categories (years of education) - N (%)**	
0-9	120 (42.4%)
10-12	116 (41.0%)
13->	47 (16.6%)
**Employment - N (%)**	
Employed	33 (11.5%)
**Marital status - N (%)**	
Single	206 (71.8%)
Other	81 (28.2%)
**Living situation - N (%)**	
Alone	103 (36.0%)
Other	183 (64.0%)
**Number of hospitalisations - mean (SD)**	**6.4 (5.9)**
**PANSS positive categories - N (%)**	
0-18	210 (73.4%)
19-31	76 (26.6%)
**PANSS negative categories - N (%)**	
0-25	217 (75.9%)
26-43	69 (24.1%)
**CDSS - mean (SD)**	3.6 (4.1)
**QoLI: Living situation - mean (SD)**	4.6 (1.5)
**QoLI: Daily activities and functioning - mean (SD)**	4.8 (1.1)
**QoLI: Family - mean (SD)**	4.5 (1.6)
**QoLI: Social relations - mean (SD)**	4.9 (1.2)
**QoLI: Finances - mean (SD)**	4.2 (1.5)
**QoLI: Legal and safety issues - mean (SD)**	4.9 (1.4)
**QoLI: Health - mean (SD)**	4.6 (1.6)
**Relapse - N (%)**	50 (17.4%)

PANSS = Positive And Negative Syndrome Scale, CDSS = Calgary Depression Scale for Schizophrenia, QoLI = Quality of Life Instrument (QoLI).

### Service use, medication, and associated costs

In Table 2, the consumption of different services and associated costs are presented for the total sample and for the subsample of service users, i.e., those patients who had used a specific service during the six months preceding the interview.

**Table 2:** Estimated service use and mean costs in total sample and among health service users during a six-month period

	**Total sample (n = 288)**		**Users of health services**		
	**Mean service use (SE)**	**Mean costs (SE) in euros**	**n**	**Mean service use (SE)**	**Mean costs (SE) in euros**
Inpatient days	5.72 (0.83)	1390 (203)	54	39.10 (3.89)	9499 (945)
Day clinic days	10.96 (1.55)	1331 (188)	49	62.35 (4.73)	7573 (575)
Psychiatrist visits	5.04 (0.23)	165 (8)	229	6.05 (0.24)	199 (8)
Psychologist visits	0.60 (0.15)	23 (6)	11	11.47 (1.67)	439 (64)
GP visits	1.50 (0.23)	25(4)	39	4.89 (0.63)	82 (11)
Other physician visits	1.32 (0.29)	29 (6)	16	8.46 (1.45)	186 (32)
Medication	-	570 (29)	280	-	579 (29)
Total	-	3534 (283)	280	-	3552 (282)

The consumption of services is presented for the total sample and for the subsample of service users, i.e., for those patients who have used a specific service.

In the total sample, the mean estimate of days spent at a day clinic was almost twice as high as the mean estimate of days spent in inpatient treatment. This trend was also noted among service users. In the total sample, visits to psychiatrists were the most numerous, followed by General Practitioners (GPs), other physicians and psychologists. Conditional upon the occurrence of at least one visit to a given category of health care professionals, psychologists were the most frequently visited.

In the total sample, a large part of the mean total cost (€ 3 534) was accounted for by the cost of inpatient treatment (€ 1 390) and day care (€ 1 331). The costs for medication ranked third (€ 570) followed by the cost for psychiatrist visits (€ 165). The total cost for the subsample of users of health services was similarly high because it was comprised of all patients used at least one service within the preceding six months, that is, almost the complete sample. The difference between the costs of inpatient treatment and day care on one hand and the other services on the other hand is even more pronounced.

The prevalence of schizophrenia in France in 2005 was estimated at 0.5% of the general population aged 15 years or older [23]. This estimate is close to the total prevalence of 0.6% reported in older studies [24]. Based on this estimate, we extrapolated from our sample the annual cost for direct medical health care for all schizophrenic patients in the country. This yielded € 621 million for inpatient treatment and € 595 million for day care, which amounts to 77.0% of the total annual direct cost (€ 1 581 million). The costs for medication (€ 255 million) accounted for 16.1% of total annual direct costs. The rest (6.9%) included visits to psychiatrists (€ 74 million), GPs (€ 11 million), other physicians (€ 12 million) and psychologists (€ 10 million).

### Cost of lost productivity

In this sample, the indirect cost linked to productivity loss for unemployed patients of working age was estimated (n = 195 patients unemployed throughout).

Given the schizophrenia prevalence in France, the annual indirect cost was extrapolated from the sample for all schizophrenic patients in the country; the estimate of the annual indirect cost of schizophrenia linked to productivity loss in France yielded € 2 214 million.

### Correlates of costs of service use and medication

Table 3 presents the estimates as found by the statistical analysis: the association of resource use costs and medication costs with socio-demographic characteristics and clinical aspects was examined by means of a between-regression model. Twenty-two percent of the variance of the total cost could be explained by the model.

**Table 3:** Correlates of direct medical mental health care costs of schizophrenia

**Variable**	**Inpatient**	**Day clinic**	**GP**	**Psychiatrist**	**Psychologist**	**Other physician**	**Medication**	**Total**
Gender (Female)	-760.49	529.15	-1.47	5.95	-4.33	-10.57	-1.45	-243.21
Age	-26.41	-18.43	0.39	-1.69	-0.19	0.84	-8.32*	-53.83
Years of education	113.54	-9.19	0.03	-2.59	1.71	-1.73	14.28	116.04
Employment (Employed)	-87.38	-1286.58*	13.92	19.03	5.81	-30.00	-16.71	-1348.48
Marital status (Single)	206.07	811.70	18.23	7.34	15.52	-2.88	64.97	1120.95
Living arrangement (Living alone)	762.76	-189.86	4.13	-5.10	21.18	-14.46	111.80	690.44
PANSS positive	141.24*	-66.16	0.03	2.75	0.38	2.60	3..88	84.72
PANSS negative	80.96	35.26	-2.54*	-1.00	1.10	-2.88*	8.69	119.58
CDSS	59.16	64.12	7.44	4.93	5.55	8.49*	24.46	174.15
SOFAS	19.33	-19.66	-0.62	-0.10	0.57	0.69	3.80	4.01
Living situation	264.66	184.65	-6.95	-24.89***	-4.42	-10.94	5.19	407.29
Daily activities and functioning	-605.17	-129.63	28.56	19.75	26.93	15.25	-24.63	-668.94
Family	129.74	79.53	-7.51	4.19	-3.70	-2.07	-5.37	205.56
Social relations	462.32	472.71	-4.65	3.28	-12.53	4.13	-27.85	897.41*
Finances	264.00	398.99*	-8.12	-2.78	10.11	3.42	26.74	692.37*
Legal and safety issues	-195.87	165.61	12.92	16.06	6.62	15.35	60.14	80.83
Health	-243.15	7.58	-5.60	-4.41	1.92	-17.80*	-3.30	-264.76
Hospitalisations	-3.61	77.02	-0.85	-0.96	-1.43	2.56	-2.45	70.28
Relapse	8443.55***	-921.47	-27.80	75.01	34.36	-68.31	619.88**	8155.22**
Constant	1020.91	737.16	12.29	156.05***	12.50	51.61**	444.48***	2435.01***
N	245	245	245	245	245	245	245	245
R² between	0.20	0.14	0.24	0.13	0.07	0.20	0.20	0.22

PANSS = Positive And Negative Syndrome Scale, CDSS = Calgary Depression Scale for Schizophrenia, QoLI = Quality of Life Instrument (QoLI).

* p ≤ 0.05; ** p ≤ 0.01; *** p ≤ 0.001.

We found that costs for day care were lower among employed patients. The higher the positive schizophrenia symptoms were at baseline, the higher the costs were for inpatient treatment during the observation period. While negative symptoms of schizophrenia were associated with lower costs for GPs and other physicians, depressive symptoms were associated with higher costs for GPs. Social and occupational functioning at baseline was unrelated to costs. There was an inverse relationship between satisfaction with living situation and costs for psychiatrists. Satisfaction with social relations was positively associated with total cost. The more satisfied patients were with their financial situation, the higher their costs were for day care and total costs. The more the patients were satisfied with their health, the lower the costs were for other physicians. There was no relationship between the number of previous hospitalisations and cost. Patients who relapsed during the follow-up period had higher costs for inpatient treatment and medication, as well as total costs, than those without relapse.

## Discussion

The cost of schizophrenia has been estimated in a number of studies. The main common result in all of these studies is the elevated hospitalisation cost, which heavily impacts direct medical mental health costs [2].

The main objective of this study was to describe and update resource allocation for French schizophrenic patients, based on the EuroSC cohort, which is a longitudinal, representative sample of French schizophrenic patients. Given the course of the disease, schizophrenic patients’ care is usually managed by their “psychiatric sectors”, French mental health catchment areas. The patient selection process allowed us to obtain a random sample of this population. Moreover, this sample was followed prospectively during a two-year period. Thus, the resource utilisation collection was standardised and prospective.

In France, in 1992, Rouillon et al. [3] were the first to evaluate the cost of treatment in medical and social terms. Because of the different methodology used, a direct comparison with this study is not possible. In the previous study, a mail survey was conducted among all practising psychiatrists in France, who were requested to provide information on the patients they had most recently seen or examined. Unfortunately, the response rate (8%) was very low, raising questions about the representativeness of the sample. By contrast, in the present study, patients were randomly drawn from three defined catchment areas located in Northern, Central and Southern France. While in the previous study the diagnosis was made by the treating psychiatrists, in the present study, it was based on a structured interview (SCID) conducted by trained interviewers. In the previous study, retrospective data on resource consumption were obtained from case files, while in the present study, patients were interviewed 5 times at 6 month intervals. In the previous study, the cost of lost employment was assessed using social assistance allowances, while in the present study, information on employment status provided by patients served as the basis for this assessment. In contrast to the previous study, costs for psychologists, GPs and non-psychiatric specialists have also been included, while costs for intermediate services other than day clinics have not been included.

As usual, the financial costs of schizophrenia to society can be divided into direct and indirect costs. In the study by Rouillon, direct costs included treatment provided in inpatient services (55%), intermediate facilities (30%), outpatient visits (9.5%) and medication costs (5.5%).

In the present study, the breakdown of direct costs was different. The proportions of costs related to inpatient care, day clinic or outpatient care were lower (39.3%, 37.6% and 6.8%, respectively) compared to the Rouillon study, and medication costs accounted for a larger part of the total costs (16.1%).

Even though the present inpatient admission cost is slightly lower than that in the Rouillon study (from 85% to 77.0%), it remained the single largest contributor to French direct costs of treating schizophrenia. The decrease in inpatient admission costs may be attributed to the cost being reported as day clinic hospitalisation.

The contribution of drug costs to the total cost of treatment seems to have increased in France (from 5.5% to 16.1%). In the study by Knapp et al. [2], the use of atypicals had pushed up drugs’ cost contribution to the total cost, partly because of their higher prices and partly because of potential reduction in inpatient stays, thus potentially reducing the total cost.

In another study by Salize et al. (2009) [25], as part of a randomised controlled trial (RCT) in six European sites, the direct mental health care cost for 422 patients with schizophrenia was analysed according to how total and medication costs differed across sites and which variables were likely to predict total or service-specific costs. A difference in the basic study design does not allow direct comparison. In the RCT, although samples were homogeneous, large inter-site cost differences were found (annual means ranging from € 2 958 in Spain up to € 36 978 in Switzerland). In this study, the annual cost was extrapolated to be € 3 796 million. In the RCT, psychopharmacologic costs were much more constant across sites than costs for other services. Total costs were associated more with regional or socio-demographic characteristics than with disorder-related parameters. By contrast, in the present study, costs were virtually unrelated to socio-demographic characteristics. The RCT confirmed remarkable differences in direct costs of patients with schizophrenia across Europe. However, the relative stability of medication costs suggested a need to analyse mechanisms that influenced service-specific costs for schizophrenia. By contrast, in the present study, we found the increase in medication costs in France to be higher and in the range of 5.5% to 16.1%. We also identified several mechanisms, such as depression at baseline, satisfaction with living conditions, social relations, cost of psychiatrists, and financial condition, that affecting the cost of treatment.

In the present study, the indirect cost linked to productivity loss for unemployed patients of working age was estimated as a heavy cost, even if the estimation was only reasonably accurate.

Several cost drivers were identified: positive or depressive symptoms of schizophrenia as well as relapse of symptoms during the follow-up period predicted higher costs; satisfaction with health or negative symptoms of schizophrenia at baseline was linked with lower costs. Socio-demographic characteristics, social and occupational functioning at baseline and number of previous hospitalisations were unrelated to costs. This finding is consistent with the results from the study by Knapp [2].

Our study has several limitations.

First, this study focuses on treatment and health care costs. Although cost of lost productivity through unemployment is included in the analysis, all other indirect costs are ignored. Costs to patients may also include personal suffering, costs associated with premature mortality (whilst premature mortality may be attributable in part to the risks of suicide, it may also be the result of other factors, such as poor living conditions, poor nutrition, or decreased access to healthcare services), costs of informal caregivers (the costs incurred by the family members of the patient incorporate several components, including personal suffering and sometimes loss of productivity that should be assessed as well), criminal justice system costs, social welfare costs, and private alternative therapy costs and may also include intangible costs.

Second, there was a high rate of attrition in the sample. Although this was handled in our statistical analysis, sensitivity analysis using several imputation techniques could provide a better understanding of the variability of the estimates.

Third, comorbidities were not taken into account in this analysis. There is evidence associating other conditions with schizophrenia that also require treatment. For example, people with schizophrenia are more likely to suffer from diabetes and cardiovascular disorders. Economic costs incurred by managing these comorbidities are and will continue to represent a considerable burden on health services [26].

Finally, although large efforts have been made to obtain a representative sample of the French schizophrenic population, we recognise that our results should be taken with caution because of the sample size.

Further research is needed on how to evaluate indirect costs and caregiver costs. Sample attrition in schizophrenia cohorts is common and appropriate techniques should be explored. Finally, comorbid illness (in general and mental health) should be included in further research.

## Conclusions

This study described resource allocation in a longitudinal, representative sample of French schizophrenic patients. Cost drivers were also identified.

In the sample of 288 French schizophrenic patients aged 18-64 years, the direct resource allocation showed that inpatient admissions remained the single largest contributor to French direct costs of treating schizophrenia, with day clinic hospitalisation and an increase in the cost of medications included as a part of inpatient admissions. Unemployment was identified as a major indirect cost of schizophrenia treatment.

Positive and depressive schizophrenia symptoms predicted a higher cost of treatment. Health satisfaction or negative symptoms of schizophrenia were linked with lower costs.

We found that schizophrenia is an economic burden in France. Based on the results obtained in France, we suggest further analysis of mechanisms that influence the service-specific costs for schizophrenia in other areas of the world.

## Competing interests

The study was funded by H. Lundbeck A/S. The authors declare that they have no competing interests.

## Authors’ contributions

ES, DH participated in the design of the study and performed the statistical analysis. ES, DH, JMA, HHK, MCA and MT conceived of the study and participated in its design and coordination and helped to draft the manuscript. All authors read and approved the final manuscript.

## Pre-publication history

The pre-publication history for this paper can be accessed here:

http://www.biomedcentral.com/1472-6963/12/269/prepub
